# Innovative Experiences at Work Support Hippocampal Maintenance in Late Life

**DOI:** 10.1093/geroni/igaa057.1307

**Published:** 2020-12-16

**Authors:** Kaleena Odd, Julie Blaskewicz Boron, Aga Burzynska, Jonathan Santo, Paul Robinson, Sherry Willis, K Warner Schaie

**Affiliations:** 1 University of Nebraska Omaha, Omaha, Nebraska, United States; 2 Omaha, Nebraska, United States

## Abstract

Prior research has demonstrated the positive impact of occupational complexity on cognitive aging, however, neural underpinnings remain unclear. There is emerging evidence linking midlife managerial experience to slower hippocampal atrophy (Suo et al., 2012, 2017), supporting the brain maintenance model (i.e. preservation of young-like brain structure). However, occupational complexity, along with education, is known to be a proxy of cognitive reserve (i.e. mind’s resistance to brain aging). The current study examined the influence of midlife work environment factors (i.e., autonomy, control, and innovation; Work Environment Scale, Moos, 1981) on change in hippocampal thickness, while controlling for education and age. We studied 150 participants (60-78 years, M = 66.27, SD = 5.20, 61% female) from the Seattle Longitudinal Study who had at least one MRI scan and remained cognitively normal between 2006 and 2014. Hypotheses were tested using multilevel modeling in Mplus; gender differences were examined. There was no substantial drop in model fit as a result of adding any of the significant effects. Innovation at work slowed the decrease in hippocampal thickness over time demonstrating the protective effect of more novelty, variety and change in work activities. There was a significant age by gender interaction, such that the decrease in hippocampal thickness was stronger for older women. Together, findings suggest that long-term impact of work environment on the hippocampus extends beyond the effects of education, particularly in men, supporting the brain maintenance hypothesis. Innovation at work should be considered in understanding protective/risk factors in hippocampal atrophy in older age.

